# Extending and Strengthening Routine DHIS2 Surveillance Systems for COVID-19 Responses in Sierra Leone, Sri Lanka, and Uganda

**DOI:** 10.3201/eid2813.220711

**Published:** 2022-12

**Authors:** Carl Kinkade, Scott Russpatrick, Rebecca Potter, Johan Saebo, Michelle Sloan, George Odongo, Tushar Singh, Kathleen Gallagher

**Affiliations:** Centers for Disease Control and Prevention, Atlanta, Georgia, USA (C. Kinkade, M. Sloan, G. Odongo, T. Singh, K. Gallagher);; University of Oslo, Oslo, Norway (S. Russpatrick, R. Potter, J. Saebo)

**Keywords:** COVID-19, respiratory infections, severe acute respiratory syndrome coronavirus 2, SARS-CoV-2, SARS, coronavirus disease, zoonoses, viruses, coronavirus, DHIS2, District Health Information Software, surveillance, Sierra Leone, Sri Lanka, Uganda, surveillance systems

## Abstract

The COVID-19 pandemic challenged countries to protect their populations from this emerging disease. One aspect of that challenge was to rapidly modify national surveillance systems or create new systems that would effectively detect new cases of COVID-19. Fifty-five countries leveraged past investments in District Health Information Software version 2 (DHIS2) to quickly adapt their national public health surveillance systems for COVID-19 case reporting and response activities. We provide background on DHIS2 and describe case studies from Sierra Leone, Sri Lanka, and Uganda to illustrate how the DHIS2 platform, its community of practice, long-term capacity building, and local autonomy enabled countries to establish an effective COVID-19 response. With these case studies, we provide valuable insights and recommendations for strategies that can be used for national electronic disease surveillance platforms to detect new and emerging pathogens and respond to public health emergencies.

In the aftermath of the 2014 Ebola outbreak in West Africa, ministries of health in the region proposed building resilient health systems capable of responding to routine health challenges and public health emergencies (*1*). In the same year, the Global Health Securities Agenda (GHSA, https://ghsa2024.org) was established to strengthen capacities to prevent, detect, and respond to public health threats (*2*). Improving reporting completeness and timeliness via electronic surveillance systems is a key tactic of the GHSA to ensure real-time data is used to target prevention activities, detect threats early, and plan response measures for disease outbreaks and public health emergencies (*3*). This report examines how 3 countries built on past investments in routine health information systems to respond to the COVID-19 pandemic.

Before the COVID-19 pandemic, many low- and middle-income countries had made substantial investments in scaling up their national health management information systems (*4*). Those efforts were often bolstered by financing from multilateral agencies or global funds, such as the Global Fund, Gavi Alliance, World Bank, and GHSA, along with US bilateral initiatives, such as the President’s Malaria Initiative and President’s Emergency Plan for AIDS Relief. In 2015, the US Centers for Disease Control and Prevention provided funds for the core District Health Information Software version 2 (DHIS2, https://dhis2.org) platform. DHIS2 is a free, open-source software platform that enables users to create data collection forms, indicators, and data visualizations. DHIS2 provides dashboard platforms to enhance capabilities for aggregate and case-based disease surveillance and learning in early adopter countries, such as Uganda and Sierra Leone (Figures 1, 2). Investments in DHIS2 resulted in functional improvements for generating predictive disease thresholds according to previously reported data and creating outbreak alerts from the system via email, short message services, or other means. During 2016–2018, dedicated regional training academies for designing DHIS2-based disease surveillance were created in Africa and Asia to enhance uptake and use of these functional improvements.

**Figure 1:**
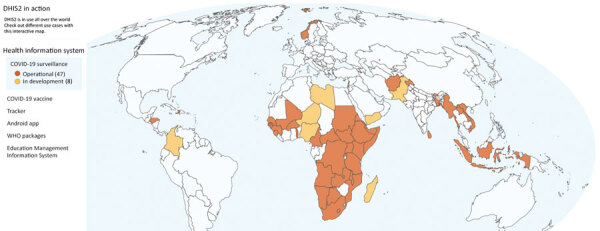
Countries using the District Health Information Software version 2 (DHIS2, https://dhis2.org) platform for COVID-19 surveillance, as described in review of extending and strengthening routine DHIS2 surveillance systems for COVID-19 responses in Sierra Leone, Sri Lanka, and Uganda. The online map (https://dhis2.org/in-action, cited 2022 Sep 8) is interactive and indicates which countries have DHIS2 operational or in development for COVID-19 surveillance in the country’s health management information system. Surveillance can include case-based surveillance, contact tracing, port of entry screening, hospital stay monitoring, call center data, and exposure risk assessment.

**Figure 2:**
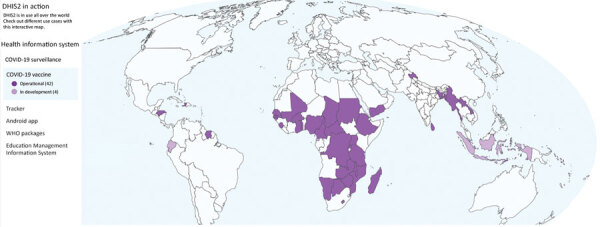
Countries using the District Health Information Software version 2 (DHIS2, https://dhis2.org) platform to monitor COVID-19 vaccination status, as described in review of extending and strengthening routine DHIS2 surveillance systems for COVID-19 responses in Sierra Leone, Sri Lanka, and Uganda. The online map (https://dhis2.org/in-action, cited 2022 Sep 8) is interactive and indicates which countries have DHIS2 operational or in development to monitor COVID-19 vaccination status in the country’s health management information system. Monitoring can include tracking electronic immunization registries, vaccine stock management, the Android Capture application, and electronic certifications.

By the end of 2019, a total of 25 countries worldwide had adopted DHIS2 as the national surveillance platform. Surveillance often began by including weekly aggregate electronic reports to the Integrated Disease Surveillance and Response (IDSR) or a similar framework for priority disease monitoring in their national health management information systems. The gradual scaling and decentralization of electronic reporting for priority diseases down to the primary healthcare level is an effort that generally takes countries years to fully achieve. Many countries began these efforts to scale up electronic reporting well before the COVID-19 pandemic, leveraging the existing DHIS2 platform at the health facility level for routine reporting in their health management information system. By 2020, each ministry of health (MOH) in >55 countries worldwide had established national DHIS2 platforms.

DHIS2 was already established globally before the COVID-19 pandemic and had extensive support structures and a growing global ecosystem of users and developers, conferences, discussion forums, and financial and technical partners. A key lesson learned from the information and communications technology response to the 2014 Ebola epidemic, reinforced during the COVID-19 pandemic, was the benefit of using existing technologies and digital infrastructure in-country to rapidly respond to an emergency. Heeding this lesson, countries including Sri Lanka, Uganda, and Sierra Leone began to adapt and configure their existing DHIS2-based systems to meet the new data collection and analysis needs for COVID-19 without establishing new parallel systems. By October 2021, a total of 55 countries had leveraged their DHIS2-based information systems to support COVID-19 detection, prevention, and response measures, including vaccinations.

This report discusses how prior investments in DHIS2-based surveillance systems by Sri Lanka, Sierra Leone, and Uganda enabled each country to rapidly respond and adapt their existing DHIS2 systems to meet the needs of the COVID-19 pandemic. Whereas emergency response measures may require innovation and novel approaches, this report shows how local innovation and self-reliance can be deployed quickly and effectively and complement existing systems and infrastructures. Furthermore, we show how local capacity and technological innovation can co-exist within existing institutionalized, national-scale deployment of DHIS2. These countries were selected because they illustrate successful outcomes of integrating emergency surveillance for COVID-19 within existing disease surveillance systems. Challenges and occasional setbacks associated with building resilient health information systems still remain. Challenges and tensions related to DHIS2 use have been reported (*5*–*7*).

## Case Studies

### Sierra Leone

Sierra Leone established DHIS2 as a national routine health information system in 2008. Concurrently, they adopted technical guidelines to implement the IDSR framework developed by the World Health Organization (WHO) Regional Office for Africa (*8*) and began comprehensive public health surveillance and response systems for priority diseases, conditions, and events at all levels of the health system. In 2016, the Sierra Leone Ministry of Health and Sanitation (MOHS), in partnership with the US Centers for Disease Control and Prevention, WHO, and e-Health Africa, began a transition from paper-based to electronic reporting of weekly aggregate IDSR data for 26 reportable diseases in public health facilities using DHIS2. National rollout was completed during 2018–2019 (*9*). Building on the success of the electronic reporting of aggregate data, the MOHS developed an electronic case-based disease surveillance (eCBDS) system for reporting individual cases from healthcare facilities to a centralized data repository. The eCBDS system was tested in 4 of 16 districts during 2018–2019 for 20 of 26 reportable epidemic-prone diseases.

Acute respiratory infection is 1 of 20 conditions being reported through the eCBDS; this condition was updated to incorporate WHO-recommended variables for COVID-19 in February 2020. By leveraging the existing electronic IDSR (eISDR) system infrastructure that included smart mobile devices, other means of accessing the internet, and trained healthcare workers, the MOHS was able to rapidly launch an integrated eCBDS reporting module for COVID-19 and other notifiable diseases in all remaining districts and healthcare facilities. To cope with fast-spreading COVID-19 and the increasing need to report data from across the country, the MOHS, with support from multiple partners, used a 3-tiered approach for eCBDS system training in the remaining districts. The tiers consisted of training the trainers at the national level, who then trained district staff, who then trained healthcare facility personnel.

In addition to case-level data reporting, eCBDS operations were enhanced to support contact tracing, quarantine monitoring, and international travel monitoring for COVID-19. The system was further expanded to integrate COVID-19 vaccination programs that included an electronic immunization registry of all persons who received vaccines. The ability to track due dates for second vaccine doses and send vaccination reminders through short message services to all eligible persons with mobile phones was also incorporated in the system. A COVID-19 vaccine adverse events reporting module was added. Platform adaptability and flexibility enabled the vaccination and surveillance data to be captured in the same system, which promoted planning for and distribution of COVID-19 vaccines. The Sierra Leone National COVID-19 Emergency Response Centre has strengthened governance of the eCBDS system since mid-2021 to ensure that useful data from various data systems and tools can be easily integrated into the eCBDS system in an emergency.

### Sri Lanka

DHIS2 was introduced in Sri Lanka in 2011 and widely used by several national health programs at the start of the COVID-19 pandemic. Before reports of COVID-19 in Sri Lanka, senior representatives at the MOH discussed the need to collect data at points of entry (POE) from travelers arriving from countries with known COVID-19 transmission as part of a prevention strategy. The Health Information Systems Program (HISP) Sri Lanka, the DHIS2 implementation group supporting the MOH, modified DHIS2 in 2 days to register all international travelers arriving through airports and actively monitor them for 14 days for potential signs or symptoms of COVID-19 infection. The director general of health services approved modifications to the system, and Sri Lanka began using DHIS2 for COVID-19 surveillance; data were added to the system beginning in January 2020 (*10*). By early February 2020, POE screening was fully functional at all airports in Sri Lanka, which enabled the country to temporarily maintain open air borders to tourists while monitoring COVID-19 globally and within the country.

Sri Lanka’s Information Communication Technology Agency (ICTA) was already experienced with hosting and supporting DHIS2; however, additional human resources were required to implement DHIS2 at POE and quarantine centers. The human resource gap was addressed by using a large pool of medical doctors who had completed a government sponsored master’s degree program in information systems and had previous experience with DHIS2. However, the need for an integrated system for all COVID-19 case reporting and surveillance data in the country quickly became apparent. Integration required new applications and DHIS2 functionalities; however, the HISP and ICTA lacked developer resources for those changes. The ICTA announced a hackathon on Twitter and recruited 25 volunteer developers, most from Sri Lanka; the University of Oslo (UiO) also loaned a DHIS2 core developer. UiO recognized that local innovations needed in Sri Lanka would likely be required in other countries. Therefore, the core developer was intended to support the development team in Sri Lanka to produce generic applications and functionality that could also be used in other countries. 

Within 2 weeks, the team of developers created a customized data capture application for POE and contact tracing data, an analytics application for COVID-19 case relationships, and an interoperability solution for exchanging data with Sri Lanka’s immigration information system. Sri Lanka also introduced a hospital bed tracking component to the COVID-19 system, permitting facility users to quickly enter and update available intensive care unit and non–intensive care unit beds. This component was invaluable in locating available hospital beds for COVID-19 patients, which enabled planning and allocation of patient flow, including to other facilities.

On January 28, 2021, Sri Lanka launched a further expansion of its COVID-19 data systems in DHIS2. Expansion included a national-scale electronic immunization registry for COVID-19, vaccine stock monitoring at vaccination sites, real-time monitoring dashboards, and interoperability with Digital Infrastructure for Vaccination Open Credentialing (DIVOC, https://divoc.digit.org) software to generate digital vaccine certificates. Interoperability solutions were used to preregister a large proportion of the population in the COVID-19 electronic immunization registry according to existing citizen registries. Government stakeholders reported that monitoring real-time vaccination rates across the country was particularly effective and contributed to rapid planning for distributing vaccine stock, which often arrived sporadically in the country with little information about vaccine quantity, type, or expiration dates. As of December 2021, a total of 19,147,151 persons in Sri Lanka were enrolled in the country’s DHIS2-based electronic immunization registry.

### Uganda

Uganda established DHIS2 as a national eIDSR system for notifiable diseases in 2013; the system included case-based reporting linked to case investigation and laboratory data for some priority diseases. At the onset of the COVID-19 pandemic, the Uganda MOH incorporated WHO-recommended data variables for COVID-19 case-based surveillance into the existing DHIS2-based eIDSR system with support from HISP Uganda.

Uganda is a hub for overland trade routes among Kenya, Tanzania, Rwanda, Democratic Republic of the Congo, and South Sudan. Continuous and essential flow of goods, especially petroleum, occurs through 60 official border crossings. Truck drivers transiting Uganda from surrounding countries elevated the risk for COVID-19 spread and faced crowded, long waits at Uganda’s borders. In response, HISP Uganda developed a new POE module within the eIDSR system to screen, test, and clear persons entering Uganda at all 60 formal border crossings. Using the DHIS2 Android Capture application, screeners at the border collected a traveler’s personal details and travel history simultaneously with specimen collection. Test samples were processed at the POE. Upon receipt of a negative test, travel clearance was provided in the form of a printed paper pass with the traveler’s photo and a quick response (QR) code. As truck drivers and passengers traveled through Uganda, they were required to present their paper passes at different checkpoints where QR codes were scanned, and the POE system automatically updated the GPS location. Truck occupants were periodically retested at checkpoints. If a driver or passenger tested positive at a checkpoint, contact tracers were able to follow up and analyze the patient’s travel history by using geographic information system tools within DHIS2.

### Global DHIS2 Community Response

Local innovation and extension of national DHIS2 systems, coordinated by UiO, inspired the development of products and guidance for DHIS2 use for COVID-19 surveillance, prevention, and response activities in 55 countries across Asia, Africa, Europe, and Latin America. HISP Sri Lanka’s POE module was shared alongside a suite of configuration packages and implementation tools for COVID-19 case-based surveillance, contact tracing, situation reports, and dashboards, following WHO technical guidance and recommendations for data collection, case definitions, and analysis. A customizable COVID-19 case-based surveillance module was made available to the global community. The design was predicated on Uganda’s and Sierra Leone’s existing DHIS2 configurations. Routine integrated case-based disease surveillance and functional requirements were identified by a global surveillance advisory group convened by WHO with support from the Gavi Alliance in 2019 for vaccine-preventable diseases. Most countries that deployed DHIS2 for COVID-19 surveillance, prevention, and response already had existing national DHIS2-based systems for some health programs. Chad, Mauritius, and Suriname adopted DHIS2 during the pandemic response. Similarly, DHIS2 developers worked closely in real-time with users to respond to emerging functional requirements, such as improved QR scanning functionality in the DHIS2 Android app and new data visualization parameters for tracking epidemic curves on dashboards.

Effects of local innovations and custom DHIS2 apps extended beyond their countries of origin and the COVID-19 pandemic. Innovations were shared in real-time through online communities of practice, webinars, informal social media chat groups among implementers and developers, and other channels to accelerate progress in other countries. Developers of the COVID-19 contact tracing app in Sri Lanka later partnered with a developer in Guinea to extend app functionality to visualize temporal transmission chains in a cluster of Ebola cases in Guinea in February 2021. HISP Mozambique used the same technology and adapted Uganda’s approach to establish a similar mobile phone integrated POE system in Mozambique and Guinea Bissau. By November 2021, dozens of countries had used QR code scanning for COVID-19 surveillance and vaccine certificates, barcode scanning for stock management and parcel tracking, and for tracking school attendance in Mozambique and The Gambia.

## Discussion

Prior investments in electronic disease surveillance systems provided a solid foundation for low- and middle-income countries to respond to the emerging data management needs for COVID-19. Through these case studies, several factors were identified that enabled rapid COVID-19 surveillance: flexible, open-source technology; communities with a strong ethos of sharing; and long-term capacity building.

The DHIS2 software is free and open source and can be customized or configured according to local requirements and adapted to changing circumstances. These features were evidenced during the pandemic by Sri Lanka’s innovative web apps for analyzing chains of transmission (*11*), Uganda’s extension of the DHIS2 Android Capture app to generate and read QR travel passes (*12*), and Sierra Leone’s rapid eCBDS configuration updates that enabled COVID-19 reporting.

Using a generic, extendable platform approach, software investments in one country can be shared, customized, reused, and ultimately translated to add substantial value in another country. Sri Lanka distributed their custom apps globally through an online DHIS2 app hub, and Uganda worked closely with DHIS2 developers to add needed functionality to the core software. In both cases, new software functionalities for COVID-19 pandemic response measures were rapidly made available to countries worldwide through continuous innovation by a diverse network of implementers, users, and developers. Developers engaged in the COVID-19 response reported that they felt a responsibility to develop generic, open-source platform extensions so that the broader DHIS2 community could benefit from their innovations, especially during a global crisis (*13*).

An inclusive and participatory community of practice enables innovations to be shared, shaped, adjusted, and improved, while also building knowledge across geographic and organizational boundaries. Sri Lanka relied on participation from independent, volunteer Sri Lanka-based developers, existing networks of master’s program alumni, and a core DHIS2 developer to create and implement novel solutions. Existing community channels have been used during the COVID-19 pandemic to assist with real-time learning and sharing, such as the community of practice web portal (https://community.dhis2.org), Health Data Collaborative (https://www.healthdatacollaborative.org) webinar series, Digital Square (https://digitalsquare.org/covid19), and the DHIS2 annual conference (https://thedac2020.sched.com). Those efforts enabled countries to learn about emerging practices, adapt solutions, make improvements, and engage with the community through the same channels.

Investments in global goods require an adequate investment in local capacity to implement and sustain these products. The UiO has supported capacity building for 3 decades by contributing to online self-study training modules, regional DHIS2 training academies, master’s and doctoral programs in low- and middle-income countries, and international exchange.

In Sri Lanka, staff with skills and experience with DHIS2 were critical for development of new COVID-19 modules and providing training for their use. Degree programs at the University of Colombo in Sri Lanka expose students to the DHIS2 platform, who can then be quickly trained on the POE module. In Uganda, a strong domestic community around DHIS2 provided the necessary capacity to develop new apps. In Sierra Leone, the institutionalization of the eCBDS and investments in governance enabled a more coherent, integrated information system supporting many aspects of the COVID-19 emergency. DHIS2-based systems around the world are not COVID-specific; rather, most are integrated health information systems that exhibit flexibility to adapt to emerging diseases and public health threats. DHIS2 can bring data together across programs for powerful analysis and use. Timely deployment of electronic surveillance systems for COVID-19 was the result of decades of decentralizing capacity to govern and manage national data systems, designing and configuring systems responsive to users’ needs, and implementing interoperable systems that achieved MOH data analysis requirements. This process illustrates the importance of system strengthening in nonemergency periods to support the needs during a public health emergency.

Countries with existing integrated case-based disease surveillance systems, such as Uganda and Sierra Leone, were able to quickly add new variables, data collection forms, and visualizations to their DHIS2 configurations. They also streamlined data collection from facilities with minimal efforts in training and rollout to meet the new COVID-19 requirements. Those countries immediately benefitted from existing electronic disease surveillance system coverage, and reporting occurred at the facility level in most districts. Local innovations were disseminated rapidly through the global community. For example, Sri Lanka pioneered the use of DHIS2 to integrate POE screening into their national surveillance system. Rather than establishing a new disease reporting system for each emerging new disease, existing systems and workflows can be modified quickly to meet new programmatic requirements.

Long-term investments in strengthening health systems contributed to core capacities for data management, information system design, and administration within different MOHs, enabling national HISP teams to rapidly modify existing electronic surveillance systems. In countries where COVID-19 surveillance data were integrated into a national system at the onset of the pandemic, key stakeholders indicated there were streamlined data flows and trust in DHIS2 as a surveillance data source. COVID-19 response funding contributed to strengthening the overall national electronic disease surveillance system in countries where COVID-19 surveillance was integrated into an existing system. In this report, we provide valuable insights and recommendations for strategies that can be used to prepare national electronic disease surveillance platforms to detect and respond to new and emerging pathogens and public health emergencies.
